# Unilateral testicular rupture after blunt scrotal trauma: A case report and literature review

**DOI:** 10.1016/j.ijscr.2025.112089

**Published:** 2025-10-21

**Authors:** Denis Mucunguzi, Donald Dominick Lema, Orgeness Jasper Mbwambo, Frank Bright, Nyamhanga Nsaho Maro, Bartholomeo Nicholaus Ngowi

**Affiliations:** a Faculty of Medicine, KCMC University, P.O BOX 3010, Moshi, Tanzania; b Department of Urology, Kilimanjaro Christian Medical Centre, P.O BOX 3010, Moshi, Tanzania; c Division of Urology, Mbarara University of Science & Technology, P.O BOX 1410, Mbarara, Uganda

**Keywords:** Blunt scrotal trauma, Unilateral testicular rupture, Scrotal haematocele

## Abstract

**Introduction:**

Testicular trauma, accounting for up to 66 % of urological injuries, mainly affects males aged 15 to 40 due to sports, violence, and traffic accidents. Rupture occurs in 48 % to 60 % of blunt injuries, often involving the right testis. Early ultrasonographic diagnosis and prompt surgical exploration are vital to prevent complications, infertility, or orchiectomy.

**Case presentations:**

A 57-year-old man presented with a three-month history of right-sided scrotal swelling following blunt perineal trauma from a fall. Initial treatment with analgesics and antibiotics from lower level health facilities relieved pain but not swelling. Examination revealed a non-tender, irreducible right scrotal mass with a non-palpable right testis. Ultrasound showed a large, mixed-echo fluid collection with increased peripheral vascularity. Laboratory results were normal. A differential diagnosis of scrotal haematoma versus abscess was made, prompting surgical exploration. Intraoperatively, a 150 mL old haematoma and a ruptured right testis (AAST grade V) with torn tunica albuginea, extruded seminiferous tubules, and necrotic tissue were found. A right orchiectomy and debridement were performed. Hemostasis was achieved, and layered closure of the scrotum was completed using absorbable sutures. The postoperative course was uneventful, and at one-month follow-up, the patient had recovered well. Early evaluation and surgical intervention were key to an optimal outcome in this case of delayed testicular rupture.

**Discussion:**

Blunt testicular trauma, although rare, requires early ultrasonographic assessment and immediate surgical exploration to maximise testicular salvage and minimise orchiectomy rates. Ruptures often occur from sports or falls, with atypical presentations making diagnosis more difficult. The AAST grading system informs treatment, and early intervention enhances fertility, hormonal function, and psychosocial outcomes.

**Conclusion:**

Testicular rupture is uncommon but serious, requiring prompt assessment to optimise testicular preservation, especially in high-risk patients.

## Highlights

• The patient experienced blunt trauma to the scrotum following a fall, resulting in persistent swelling on the right side of the scrotum for three months.
• Initial treatment with analgesics and antibiotics alleviated pain but did not reduce the swelling, leading to a referral to a higher-level facility.
• Examination revealed a non-tender, irreducible, non-transilluminant right scrotal mass with a non-palpable right testis.
• Ultrasound showed a large, heterogeneous fluid collection suggestive of a haematoma, raising suspicion of an abscess or testicular rupture.
• Surgical exploration confirmed a ruptured right testis (AAST grade V) with necrosis, resulting in orchiectomy and debridement, followed by an uneventful recovery.

## 1 Introduction

Approximately 33 % to 66 % of all urological traumas involve genital injuries, which are more common in men due to anatomical differences, higher risk of traffic accidents, and increased participation in contact sports, conflicts, and criminal activities [1]. The three most frequent causes of blunt testicular trauma are sports injuries, interpersonal violence, and traffic collisions.

Blunt testicular trauma mainly affects males aged 15 to 40 [2]. Due to the anatomical position and mobility of the scrotum, scrotal trauma accounts for less than 1 % of all trauma-related injuries [3]. Ruptures occur in about 48 % of cases of blunt testicular trauma. In routine urological practice, acute trauma-induced testicular rupture is uncommon [3].

Testicular trauma can cause serious complications, including rupture in up to 48 % to 60 % of cases [1,4]. Although it is not usually fatal, early detection and prompt treatment are vital to prevent adverse outcomes [5]. A tear or rupture in the tunica albuginea, leading to extrusion of seminiferous tubules, is the main sign of testicular rupture, which is rare but can be serious. The right testis is more often injured because it is more likely to be anchored against the pubis or inner thigh [6].

Testicular trauma is the third most common cause of acute scrotal pain [7]. Studies show that rupture requires a direct force of about 50 kg, with damage ranging from minor lacerations with minimal extravasation to destruction of the parenchyma [6]. These injuries often present as large, painful, ecchymotic scrotums, which can complicate clinical examination. Failing to detect a testicular rupture can lead to serious consequences [7].

Although rarely life-threatening, loss of a testicle can affect self-confidence, cause hypogonadal symptoms, and impair future fertility [8]. When a haematocele accompanies testicular injury, scrotal ultrasonography improves the physical examination. Therefore, early assessment is essential for both diagnosis and treatment.

Reducing the time between injury and surgical exploration is crucial [9]. Over 15 years, four out of fifty-three patients with haematocele following testicular trauma showed bilateral involvement. It has been proven that conservative management often results in unsatisfactory outcomes, with a high orchiectomy rate of 45 % and delayed exploration in 40 % [9]. This case report has been reported in line with the SCARE 2025 checklist [10].

## 2 Case presentation

A 57-year-old man presented with a right-sided scrotal swelling that has persisted for three months. This followed a fall while climbing a ladder, during which he sustained a blunt injury to the scrotum from direct impact on the perineal region. He described an acute, sharp, localised scrotal pain that was followed by swelling and increased warmth around the scrotum. Review of other systems was unremarkable.

He initially sought treatment at a nearby health facility and was prescribed painkillers and antibiotics before being discharged home. One month later, he noticed the pain had ceased; however, the swelling remained, and he was advised to seek assistance at a higher-level facility.

**Fig. 1:**
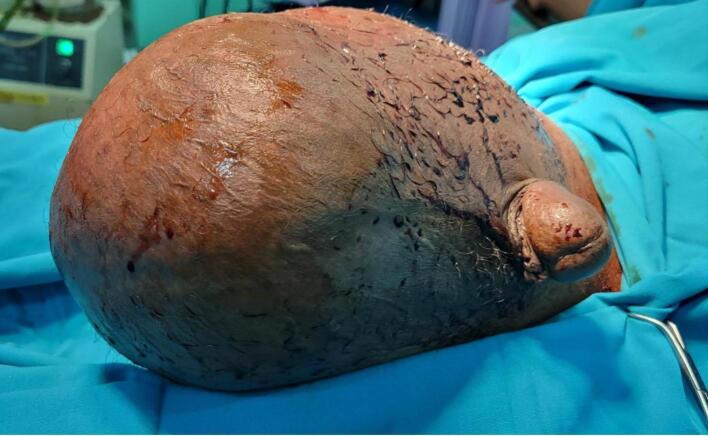
shows the large swollen scrotum, painted with povidone‑iodine and draped shortly before surgery began.

On examination, he was clinically stable, and his vital signs were within normal limits. The abdomen was soft with a normal contour. The scrotum was markedly swollen and asymmetrical (Fig. 1), with the right side more swollen than the left, having normal overlying skin, no visible scars, and non-transilluminant. It was possible to palpate beyond the swelling, which was irreducible and non-tender. The right testis could not be palpated, but the left hemiscrotum appeared normal. The rest of the systemic examination was unremarkable. The patient had laboratory work done, and the test results are presented in a table (See Table 1.)

**Table 1:** Shows the laboratory test results for the patient.

Parameter	Results	Flags	Ref. Interval
Serum creatinine	104 μmol/L	Normal	62–106
Estimated GFR	68 mL/min/1.73m^2^	Mildly decreased	≥90
Serum Sodium	132.30 mmol/L	Low	136.00–145.00
Serum potassium	4.90 mmol/L	Normal	3.50–5.10

Full Blood Count			
Leucocyte Count H]	6.49 × 10^9^/l	Normal	4.00–11.00
Erythrocyte Count3	5.50 × 10^12^/L [L]	Normal	4.60–6.50
Haemoglobin]	15.0 g/dl	Normal	13.0–18.0
HCT	46.9 %	Normal	40.0–54.0
MCV	85.4 fL	Normal	80.0–100.0
MCH	27.2 pg	Normal	27.0–32.0
MCHC	31.9 g/dL	Normal	32.0–36.0
RDW	14.0 %	Normal	11.0–16.0
Platelet Count	219 × 10^9^/L	Normal	150–500
Mean Platelet Volume	11.2 fL	Normal	6.0–11.0
Platelet Distribution Width	16.3 %	Normal	11.0–18.0

Differential			
Neutrophils	67.4 %, 4.17 × 10^9^/L	Normal	2.00–6.90
Lymphocytes	27.1 %, 1.73 × 10^9^/L	Normal	0.60–3.40
Monocytes	4.2 %, 0.07 × 10^9^/L	Normal	0.00–0.90
Eosinophils	0.6 %, 0.49 × 10^9^/L	Normal	0.00–0.70
Basophils	0.7 %,0 0.03 × 10^9^/L	Normal	0.00–0.20
Blood Grouping and cross match (ABO + RH GROUP)	ABO Group = A, Rhesus (D) Positive		
Urine Analysis	Normal finding		

Scrotal ultrasonography showed a large fluid collection with mixed internal echoes and increased blood flow in the surrounding tissues on colour Doppler. The swelling was compressible and did not communicate with other structures. Full blood count, serum creatinine, and electrolytes were all within normal ranges. The differential diagnosis of a scrotal hematoma to exclude an abscess was made; the patient consented and was prepared for scrotal exploration.

**Fig. 2:**
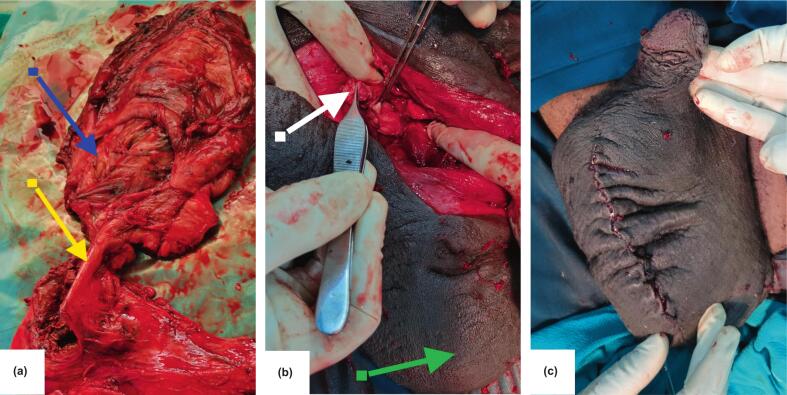
shows the tissue after orchiectomy, with part of the spermatic cord **(a, yellow arrow)**, ruptured right testis, and surrounding fibrotic tissues **(a, blue arrow)**. The stump of the spermatic cord **(b, white arrow)** after right orchiectomy, and the grossly normal left testis within its hemiscrotum **(b, green arrow)**. The closed scrotal wound **(c)**. (For interpretation of the references to colour in this figure legend, the reader is referred to the web version of this article.)

Intraoperatively, an old haematoma measuring 150 mL was found in the right hemiscrotum and was evacuated. We observed a ruptured right testis (AAST grade V injury) with a torn tunica albuginea, significant tissue loss, and extravasation of the seminiferous tubules. The tunica vaginalis appeared thick and fibrotic, with surrounding necrotic tissues in the scrotum (Fig. 2a). The left testis was palpably normal.

A right orchiectomy was performed, along with debridement of the necrotic tissues. The stump of the spermatic cord was ligated with Vicryl 1, and hemostasis was achieved (Fig. 2b). The scrotum was closed using Vicryl 2/0, and the skin was repaired with Vicryl 3/0 absorbable stitches placed in an interrupted manner (Fig. 2c). The postoperative period was unremarkable, and at follow-up after a month, the patient was doing well.

## 3 Discussion

Because blunt testicular trauma is a rare event, it requires prompt diagnosis and immediate surgical intervention [11]. Most testicular ruptures result from sports-related injuries, often due to being struck in the groin, but falls and straddle injuries can also be causes. However, according to a review of the literature, there have been five cases of rupture linked to testicular malignancies, the most recent documented in 2014 [2].

Rahul and colleagues reported a case of an incidental testicular tumour discovered after surgical exploration of a severed testis following a testicular injury caused by trivial blunt trauma [3]. Patients with testicular rupture may initially exhibit very general symptoms such as scrotal swelling and severe pain, but the characteristic signs and symptoms include oedema, ecchymosis, chronic pain, irregular testicular position, and abnormal testicular contour [11].

The differential diagnosis includes epididymitis, testicular fracture, testicular torsion, dislocated testes, hydrocele, or haematocele. However, as seen in our patient, rupture may be present with little to no pain, which can alter clinical examination and triage of injury [11]. High-frequency ultrasonography (US) with a linear-array transducer is the preferred method for assessing patients with acute scrotal pain following trauma. It is valuable in determining which patients should be treated medically or surgically because it accurately detects testicular contusions, intra- and extratesticular haematoma, and ruptured tunica albuginea [7].

Testicular rupture in high-risk patients cannot be reliably diagnosed using ultrasonography. However, it is always associated with an abnormal ultrasound, even if the findings are often ambiguous. In cases involving high-energy transfer mechanisms, maintaining a high level of suspicion is crucial. For high-risk patients, prompt investigation is essential and significantly improves the chances of saving the testicle [12].

Given the high incidence of testicular ruptures associated with scrotal trauma, early detection and surgical intervention are crucial for achieving a high salvage rate. Our case findings highlight a significant delay in referral from lower-level healthcare facilities, with an average three-month lag before the patient reached specialized care and this delay may contribute to worsened outcomes. Strengthening referral systems and early recognition of testicular pathology at first-contact facilities represent key opportunities to improve patient outcomes [8]. Conservative management of testicular contusions or ruptures resulted in a 45 % failure rate and a 45 % orchiectomy rate when delayed exploration was required among the 66 male patients treated for testicular injury [4].

The severity of testicular injury is graded using the American Association for the Surgery of Trauma (AAST), which classifies injuries from grade I to grade V. Grade I involves a contusion or haematoma; Grade II is a subclinical laceration of the tunica albuginea; Grade III is a laceration of the tunica albuginea with less than 50 % parenchymal loss; Grade IV is a major laceration of the tunica albuginea with 50 % or greater parenchymal loss; and Grade V involves total testicular destruction or avulsion. This grading system helps determine the appropriate treatment approach, with Grade I & II injuries typically managed with exploration and repair, Grade III & IV may require exploration with partial orchiectomy or orchiectomy, and Grade V injuries usually necessitate orchiectomy [13].

The American Urological Association recommends immediate scrotal exploration in all patients suspected of testicular rupture to prevent testicular loss, infection, chronic pain, infertility, and altered self-image [5]. Patients with testicular trauma who are examined early experience a shorter hospital stay, less impairment, a quicker return to normal activities, and an orchiectomy rate of just 9 % [4].

To preserve the testicle, shorten convalescence and disability, and potentially maintain fertility and hormonal function, it is advised that boys with hematocele, intratesticular haematoma, or heterogeneous testicles undergo urgent surgical assessment [14]. About 44.6 % of testicular injuries result from blunt trauma, and 48.3 % require subsequent scrotal or testicular surgery. Among these, 37.3 % had repair of a scrotal or testicular laceration, followed by unilateral orchiectomy in 23.4 % of cases, based on a descriptive analysis of scrotal and testicular trauma in the USA [5].

Although abnormal semen analyses and atrophic testes following testicular trauma clearly indicate subfertility, the condition does not seem to be immune-mediated, and patients do not necessarily exhibit infertility. However, early repair can help preserve both fertility and hormonal function [15]. Follow up is important though in our case, we were limited by a short follow-up period which limits our ability to draw firm conclusions about long-term testicular function and fertility outcomes however when a substantial loss of testicular tissue is likely, urologists should consider fertility options and offer locally accessible fertility preservation methods [1].

## 4 Conclusion

Testicular rupture is an uncommon but serious injury, and both symptom management and preservation of the testicle depend on prompt assessment and treatment. For a small percentage of adolescent boys with testicular rupture, a conservative approach may resolve the injury and maintain the testicular architecture. In patients who are at high risk, early investigation is crucial and offers a substantial chance of testicular preservation.

## Consent

Written informed consent was obtained from the patient for publication of this case report and accompanying images. A copy of the written consent is available for review by the Editor-in-Chief of this journal on request.

## Ethical approval

We received a waiver from the KCMC Institutional Review Board.

## Funding

No funds were received for this work.

## Author contribution

**Denis Mucunguzi:** Writing the original draft, review & editing, resources, project administration.

**Donald Dominick Lema:** Writing-review &editing, resources.

**Orgeness Jasper Mbwambo:** Writing-review& editing, supervision.

**Frank Bright:** Supervision, resources.

**Nyamhanga Maro Nsaho:** Resources, conceptualization.

**Bartholomeo Nicholaus Ngowi:** Supervision, Writing-review & editing, conceptualization.

## Guarantor

Denis Mucunguzi.

## Research registration number

Not applicable

## Conflict of interest statement

The author declares that there are no competing interests.
